# Consensus, Global Definitions of Whole Grain as a Food Ingredient and of Whole-Grain Foods Presented on Behalf of the Whole Grain Initiative

**DOI:** 10.3390/nu14010138

**Published:** 2021-12-29

**Authors:** Jan-Willem van der Kamp, Julie Miller Jones, Kevin B. Miller, Alastair B. Ross, Chris J. Seal, Bin Tan, Eleanor J. Beck

**Affiliations:** 1 The Netherlands Organization for Applied Scientific Research (TNO), Microbiology and Systems Biology, 3704 HE Zeist, The Netherlands; 2 Department of Family, Consumer & Nutritional Sciences, St. Catherine University, St. Paul, MN 55105, USA; jmjones@stkate.edu; 3 General Mills, Bell Institute of Health and Nutrition, Minneapolis, MN 55427, USA; kevin.miller2@genmills.com; 4 AgResearch, 1365 Springs Road, Lincoln 7674, New Zealand; alastair.ross@agresearch.co.nz; 5 Human Nutrition Research Centre, Public Health Sciences Institute, Newcastle University, Newcastle upon Tyne NE2 4HH, UK; chris.seal@ncl.ac.uk; 6 Academy of National Food and Strategic Reserves Administration, Beijing 100037, China; tb@ags.ac.cn; 7 School of Medicine, University of Wollongong and Illawarra Health & Medical Research Institute, Wollongong, NSW 2522, Australia; eleanor_beck@uow.edu.au

**Keywords:** whole grain, whole-grain food, definition, ingredient, labelling

## Abstract

Proposed global definitions of whole grain as an ingredient and whole grain food are presented by the authors on behalf of the Whole Grain Initiative. Whole grains are an important pillar of healthy and sustainable diets. Internationally accepted credible definitions of whole grains as food ingredients and whole-grain foods are necessary to ensure that all global stakeholders have shared standards, and that consumers find them clear, credible, and useful. Based on widely accepted, existing definitions and new developments, the Definitions Working Group of the global Whole Grain Initiative, with experts from academia, government agencies and industry, developed definitions for global application. The key statements of the definition documents are as follows: “Whole grains shall consist of the intact, ground, cracked, flaked or otherwise processed kernel after the removal of inedible parts such as the hull and husk; all anatomical components, including the endosperm, germ, and bran must be present in the same relative proportions as in the intact kernel” and “A whole-grain food shall contain at least 50% whole-grain ingredients based on dry weight. Foods containing 25–50% whole-grain ingredients based on dry weight, may make a front-of-pack claim on the presence of whole grain but cannot be designated ‘whole grain’ in the product name”. The definition documents have been ratified by the leading international scientific associations in this area. We urge that these consensus Whole Grain Initiative definitions be adopted as the basis for definitions used by national regulatory authorities and for health promotion organisations worldwide to use in nutrition education and food labelling.

## 1. Introduction

### 1.1. Whole Grains—Dietary Recommendations, Rationale, and Intake

Increased intake of whole grains in population studies is consistently associated with a lower all-cause mortality and with reduced risk of lifestyle-related diseases such as cardiovascular diseases, type 2 diabetes and colon cancer [1,2]. Consequently, consumption of whole grains is recommended in dietary guidelines worldwide [3,4]. Recommendations range from generic advice to include eating a variety of grain-based foods, mostly whole-grain and/or products high in cereal fibre, to more quantitative recommendations. In the United States, dietary guidelines advise to consume at least 3 servings/day, equating, for example, to 3 slices of whole-grain bread containing approximately 48 g whole grain, in order to make half your grains whole grains [5]. In Denmark, an intake of at least 75 g whole grains/10 MJ is recommended. Sweden recommends 70 g and 90 g of whole grains for women and men, respectively [6]. Because nearly all people in every country include one or more varieties of cereal grain in their diet, substituting whole grains for refined grains should be less challenging than introducing new a new food group into the diet, and an important step for improved public health (or reduced rates of non-communicable diseases).

In contrast, average dietary intake of whole grains in almost all countries is well below recommended levels, thus the fibre, nutrients and phytonutrients they contain are not part of the diet [7,8,9,10,11,12]. This deficit has the potential to contribute to chronic diseases and to their attendant high health costs, which must be shouldered by individuals and governments [13,14,15].

Increased intake of whole grains is also aligned with the shift to plant-based diets occurring throughout the world, outlined in recommendations for healthy and sustainable eating patterns. Guidelines for sustainable diets tend to suggest a higher intake of whole grains than is currently recommended by national organisations. For example, the EAT–Lancet Commission on healthy diets from sustainable food systems recommends an intake of 232 g whole grains/day—corresponding to 11 servings/slices of bread [16]. Increasing the consumption of fruits, vegetables and whole grains and decreasing red meat consumption remain key population objectives of many national dietary guidelines. A modelling study on healthfulness and sustainability of national and global food-based dietary guidelines indicated that healthy, mainly plant-based diets from sustainable food systems have a major impact on sustainability and contribute to a larger extent to health benefits than the diets recommended in current national dietary guidelines [17]. This emphasises the need for, and urgency of, actions that contribute to an increased intake of whole grains.

### 1.2. Trends in Consumption, Consumer Perceptions, and Desires for Labelling

The market for whole-grain foods and ingredients is growing worldwide. Growth is noted for a number of formerly less well-known grains, including quinoa, amaranth, spelt, teff and millet, although their consumption remains minor compared with the major cereal grains of wheat, rice and maize. Manufactured products with whole grains are increasingly entering new markets, such as Southeast Asia and Latin America. Due to this trend, Malaysia [18] and Brazil [19] recently published definitions for whole-grain foods.

Whole-grain consumption remains, on average, significantly below recommended levels, despite the growth in availability of whole-grain foods. Although the taste of whole-grain products may be appreciated by more consumers over time, raising the content of whole grains in products may result in a lower appreciation by some. This is particularly true when consumers are not familiar with the taste and colour of whole grains. In order to capitalise on the positive image of whole grains, some food manufacturers may highlight the presence of low levels of whole grains through on-package images or textual messages front-of-pack. Although such statements may be factually correct, a study showed that consumers may misinterpret such messages as signifying the presence of higher amounts of the highlighted ingredient than is actually present [20]. For example, consumer organisations in Europe and Brazil expressed the need for high levels of whole grains and non-misleading labelling for whole-grain foods front-of-pack [21,22]. In recent years, front-of-pack labelling has been recognised by the WHO as an important policy tool for promoting healthy diets and preventing obesity and diet-related non-communicable diseases [23]. As a result, it has become a key issue in debates on regulations for nutrition and health-related labelling.

### 1.3. The Need for Globally Accepted Definitions for Whole Grains as a Food Ingredient and for a Whole-Grain Food

At the 6th International Whole Grain Summit, Vienna, 13–15 November 2017, key goals and actions were identified to contribute to an increased intake of whole-grain products. To carry out the action points, the Whole Grain Summit participants agreed to work together in the global “Whole Grain Initiative” [24]. The Whole Grain Initiative gathered an international working group on definitions, with over 40 expert members from academia, government agencies and industry from Asia, Europe, North and Latin America, Oceania and Africa, to realise the first goal—“*to reach consensus on a global definition of whole grain (as raw material) and on the definition of a whole-grain food*”. The group considered widely accepted definitions as well as new developments in breeding, processing technologies, and markets worldwide as well as the needs and wishes of consumer organisations with regard to whole grain-related regulations and labelling. Actions by regulatory authorities currently developing their own whole-grain definitions were also included in deliberations.

The development of the global Whole-Grain Food definition with input from stakeholders from around the world is not intended to denigrate refined or enriched refined grains in any way. Data from nationally representative dietary intake surveys consistently shows that consumption of whole grains is well below recommendations, despite reported health benefits of higher whole-grain intake. It is recognised that dietary guidance may include allowances for enriched grain intake, but because enriched and refined grain intake is not a shortfall in the diet, the focus of this definition is to promote whole grains. The whole-grain food definition described in this paper serves multiple purposes, but, ultimately, all are intended to promote public health and consumer confidence.

## 2. The Global Definition of Whole Grains as a Food Ingredient 

### 2.1. General Remarks

Definitions of whole grains as a raw material and a food ingredient are included in food regulations in many countries. The existing definitions are aligned, namely that whole grains shall consist of the intact or processed edible components of the kernel, including the endosperm, germ, and bran, which must be present in the same relative proportions as in the intact kernel [25,26]. However, differences exist regarding the grains included and allowed processes. The core statement of the proposed definition is shown in Box 1. The full definition document is included in the Supplementary Materials (Document S1). The key points of the definition—the meaning of the terms kernel, endosperm, bran and germ, the grains to be included and processing aspects—are outlined below.

Box 1 **Whole Grain Initiative—Definition of Whole Grain as a Food Ingredient.**

Whole grains shall consist of the intact, ground, cracked, flaked or otherwise processed kernel after the removal of inedible parts such as the hull and husk. All anatomical components, including the endosperm, germ, and bran must be present in the same relative proportions as in the intact kernel.

In cereal science and technology, and in milling, the bran includes the aleurone layer, whereas in botanical definitions the aleurone layer is considered to be part of the endosperm. The term kernel in the definition is used for many widely consumed grains, such as wheat, maize, rice, barley and rye. Other commonly used terms include seed, berry, groats and simply ‘grain’. Additional terms, both in English and other languages, may be used as well. As stated in the definition, the use of the term wholemeal may be legally protected in some jurisdictions and may be equivalent to whole grain. The use of the term wholemeal versus whole grain should be checked within local contexts.

### 2.2. Grains to Be Included

All currently accepted definitions include cereal grains of the *Poaceae* grass family and, in many cases, also selected pseudocereals. Pseudocereals are defined as fruits or seeds of non-grass species that are consumed in a similar way to cereals because their nutritional profiles, preparation and uses are similar to cereal grains. Most definitions do not specify in detail the species of grains included. The definition of the Cereals & Grains Association (C & G Association, formerly AACC International) provided a list of the cereal grain species with all edible cereal grains known at that time, as well as the following three widely used pseudocereals: amaranth, buckwheat and quinoa [25]. Considering that the whole grains of all these species contain higher levels of dietary fibre and other beneficial compounds than their refined counterparts, and taking into account the benefit of global harmonisation of whole-grain definitions, the same broad range was adopted in a previous collaborative definition developed by the Healthgrain European Union Integrated Research Project, with input from a large number of universities and industries in Europe [26]. Oilseeds, pulses and legumes differ substantially from cereal grains in their anatomy and composition, and are not included in any definitions nor in dietary recommendations for whole grains [3].

Some other organisations do provide similar lists showing examples of sources of whole grain [27,28]. In Denmark and Sweden, evidence of health benefits of specific grain species is required. Therefore, only wheat (including spelt), rye, oats, barley, maize, rice, millet and sorghum are listed as whole grain. The consensus global definition presented here allows for the addition of newly developed species of cereal grains, when they are accepted by the relevant authoritative groups. For example, the recently launched Tritordeum species obtained by crossing durum wheat with the wild barley *Hordeum chilense,* is currently grown and included in products sold in Australia and Europe. Such developments through breeding can improve both nutrient content and consumer acceptance, and these grain species should be included in whole grain definitions [29].

In dietary guidelines, pseudocereals are usually included in the grains and grain product categories. Amaranth, buckwheat, (including both common buckwheat and tartary buckwheat) and quinoa are mentioned in a number of definitions and are the most common pseudocereals consumed. Their inclusion also increases the number of gluten-free whole-grain options available for individuals with coeliac disease. These ‘established’ pseudocereals are listed in the Annex of the global definition. Including the pseudocereals into the Annex allows for the addition of new pseudocereals without changing the definition.

The pseudocereal area is dynamic and the working group was required to consider seeds which are sometimes called whole grains. For example, while chia is often mentioned, its nutrient profile with ~40% dietary fibre is, contrary to amaranth, buckwheat and quinoa, far outside the range found in cereal grains [30]. Two other seeds were suggested for inclusion, djulis and jitoumi. Djulis (*Chenopodium formosarum*) is also called red quinoa and is related to quinoa (*Chenopodium quinoa*), but currently produced at a very small scale [31]. Jitoumi (*Euryales Semen*, *Euryale ferox*) are the seeds of the pricky waterlily, currently used in parts of India and China as food, but detailed compositional and health-related data are currently not available. Considering all available information, it was concluded that there are currently no convincing arguments for addition of grains other than the three pseudocereals currently included in the Annex. As more innovation in the area emerges, the Annex may be updated.

The Annex also includes: “The anatomical components of pseudocereals, being dicotyledons, are different from those of the monocotyledonous cereal grains. As for cereal grains, all edible anatomical parts of processed pseudocereals must be present in the same relative proportions as in the intact seed”.

### 2.3. Processing Aspects 

All existing whole grain definitions require that the edible components of the grain are present in their original proportions when the grains have been processed. Most grains need to be processed before consumption, which may include steps such as cleaning (e.g., removal of stones, stems, etc.), removing inedible parts (e.g., hull/husk), and both dry (e.g., milling) and wet (e.g., malting, sprouting, fermenting) processing to make the grains more stable for storage, palatable, and often with the side effect of improving nutrient bioavailability.

In most commonly applied milling processes, endosperm, bran and germ fractions are separated for later recombination. When a long shelf life is required, the germ and bran fraction are usually heated to help stabilise and reduce rancidity, followed by recombination with the endosperm from a different batch of grain. In most large flour milling plants, a wide range of varieties of the same grain species are processed and grains from these varieties may be intermingled before or during processing. Therefore, the endosperm, bran and germ of the recombined whole-grain flour may originate from different varieties or at least batches.

Many producers of consumer products also practice ‘recombination’—then usually called reconstitution—where the various fractions are recombined to the specified proportions at their point of use, compared with at the mill. Some national regulations, including from Denmark and Spain, do not allow reconstitution instead of recombination at the mill. In discussions in and outside the Working Group, proponents of this restriction mentioned that consumers may consider reconstituted whole-grain flour as inauthentic. However, reconstitution may result in lower costs and may create opportunities for improving taste and texture. For example, bakeries in the EU HealthBread project recombined the softened bran fraction after a long pre-fermentation [32]. Both recombination and reconstitution, as well as other new processes, are allowed by the consensus definitions provided that manufacturing practices to ensure quality are applied. The goal is safe and taste-acceptable whole-grain products created from processes not limited to grinding, cracking and flaking mentioned in previous definitions of whole grain. For fermentation, malting, and sprouting, the definition presented here has adopted the conditions set by C & G Association [25] and the Healthgrain Forum [33], which stipulate nutrient values have not diminished and—for malting and sprouting—that the length of the sprout should not exceed kernel length.

Mycotoxins, agrochemicals, and microbial contaminants tend to be concentrated in the outer pericarp layer. The option for removal of a minor part of the grain kernel is included in some definitions (Healthgrain [26], Germany, Switzerland, and Denmark). The Healthgrain definition states “*small losses of components—that is, less than 2% of the grain/10% of the bran—that occur through processing methods consistent with safety and quality are allowed*”. Considering the large variations that might occur world-wide regarding the specific grain type or variety, and local regulations or constraints, no quantitative limit is included in the global definition, but—as stated in the definition document (Supplementary Document S1): “*allowable limits for the percentage removed should be evidence-based and should be kept to a minimum*”.

## 3. The Global Definition of a Whole-Grain Food

### 3.1. General Remarks

Definitions of traditional whole-grain foods, such as bread, pasta and biscuits, have been in place already for many years in a number of countries. Current definitions, however, lack consistency. For example, amongst countries, the minimum percentage of whole-grain flour required for labelling bread as whole grain varies between 50 and 100% of the flour. For biscuits and pasta products, similar variations can be found [33]. Consumer acceptability of products with high levels of whole grains has limits and may be a reason why manufacturers choose lower levels of whole grains in a number of foods.

In response to the growing interest in mentioning ‘whole grain’ on food labels of a wide range of products, the C & G Association proposed a first generic definition in 2013, as follows: *a whole grain food product must contain 8 grams or more of whole grain per 30 grams of product* [25]. In 2017, the Healthgrain Forum proposed the following definition with a similar minimum level of whole grain: ≥30% whole-grain ingredients, but on a dry-weight basis, enabling products with a high moisture content to be defined as a whole-grain product. Products required more whole-grain than refined-grain ingredients [33], setting an effective threshold of >50% for products based on 100% cereal flour. The latter addition was based on the current definition in Denmark [34] and concerns expressed by the European Consumer Organisation Bureau Européen des Unions de Consommateurs (BEUC), the umbrella group for 46 independent consumer organisations from 32 countries in Europe [13]. New generic definitions were issued in Malaysia in 2020 [18] and in Brazil in 2021 [19], and are being discussed in a range of other countries. The publication of a global consensus definition endorsed by leading international scientific associations in this area may contribute to harmonisation of definitions world-wide. Box 2 shows the core statements of the Whole Grain Initiative definition. The full definition document is included in the Supplementary Materials (Document S2).

Box 2 **Whole Grain Initiative Definition of a Whole-Grain Food**

**I.**  **Definition of a whole-grain food**

A whole-grain food shall contain at least 50% whole-grain ingredients based on dry weight

**II.**  **Requirements for designating the presence of ‘whole grain’ front-of-pack**

Foods containing a minimum of 25% whole-grain ingredients based on dry weight, may make a front-of-pack claim on the presence of whole grain but cannot be designated ‘whole grain’ in the product name. *

* The decision to include “and at least 8 g/serving” in addition to “a minimum of 25% whole-grain ingredients based on dry weight” should be left to national authorities.

### 3.2. A Generic Definition Based on Dry Weight 

There is a growing array of whole-grain products, but they vary widely in their proportion of whole-grain ingredients. Grain-based food products have moisture levels varying from less than 5% for dry biscuits to about 90% for grain-based alternatives for milk and yoghurt. 

Unfortunately, regulation on labelling is limited or absent in some jurisdictions and labelling of whole-grain content of food is further complicated by the wide variety of foods and particularly the amount of water present in the final product. The amount of water in a whole-grain product is important because it impacts the overall weight, making it hard to directly compare the amount of whole grains in a dry product compared with a wet or cooked product, if water content is not taken into account. Therefore, a generic definition, based on setting a minimum level of whole grains on a dry-weight basis, was considered as the most realistic option for the global definition. 

As stated in the definition, the dry weight of a food or ingredient is the weight of the food after its moisture content has been subtracted from its total weight. The content of whole grains is the dry weight provided by all whole-grain ingredients expressed as a percentage of the total dry weight of the food product. This percentage (to be used for the definition) may be based on analysis, or on calculations with accepted data for the ingredients, such as presented in food composition databases.

The percentage of whole grains to be designated on the pack should be based on local regulations, such as the widely used Quantitative Ingredient Declaration system (QUID), also included in the Codex Standard 1, 1985, Section 5.1: Quantitative ingredients declaration [35]. For products with a high moisture content, the percentage of whole grains based on dry weight will be higher than the percentage based on QUID. This practice—calculation of the percentage of whole grains for the definition based on dry weight and calculation for labelling according to the QUID system—has already been followed for over 20 years in Denmark and Sweden [28]. An example of these calculations is presented in the Supplementary Materials (Document S3).

### 3.3. A Whole-Grain Food—At Least 50% Whole-Grain Ingredients Based on Dry Weight

In the Healthgrain Forum definition, at least 30% whole grain on a dry weight basis is required, and levels of refined-grain ingredients should not be higher than those of whole-grain ingredients. However, with the Healthgrain definition, a whole-grain product may contain on a dry-weight basis 30% whole grain and, for instance, 70% starch. In the currently presented consensus definition, with at least 50% whole grains, the sum of whole-grain ingredients will be the majority of the product on a dry-weight basis. This is important to minimise the potential for consumer confusion. Foods with a low percentage content of whole grains cannot be labelled as a whole-grain food. It is recognised that ≥50% whole grains may be difficult for products containing high amounts of other ingredients, such as pizza with toppings and some bakery products. However, given the aim of the project is to encourage whole-grain intake to improve health outcomes, it may be argued that such foods should not be considered “whole-grain”, and this may encourage reformulation of current products to meet higher standards. Previously, gradual changes in composition have been applied successfully [36]. As outlined in detail below, foods containing a minimum of 25% whole-grain ingredients based on dry weight, may make a front-of-pack claim on the presence of whole grains. For foods with low levels of other ingredients, such as bread and pasta the ≥50% threshold is below the (near) 100% level of whole grains required in many national regulations.

In line with our aim of stimulating the intake of whole grains, the definition document states that national regulations and definitions, which require a greater proportion of whole grains in a product, will prevail, whereas in countries with existing definitions that permit less than 50% for labelling a product as a ‘whole-grain food’, a change in regulations and the adoption of the proposed definition is strongly encouraged.

In whole-grain foods, especially those with high levels of grains, the percentage of whole grains may vary considerably; when only grains are present, a whole-grain food may contain at least 50%, and up to 100% whole-grain ingredients based on dry weight. Reporting the percentage of whole grain in a product in any front-of-pack labelling is strongly recommended in the definition, for ensuring fair practices in the food trade and ease of consumer comparison among and between products. This is required in the recent definitions in Malaysia [18] and Brazil [19].

### 3.4. Minimum Level for Mentioning Whole Grain Front-of-Pack: 25% Whole-Grain Ingredients Based on Dry Weight

The presence of whole grains in a product is often highlighted with pictures of grains as part of front-of-pack labelling. Such pictures have been criticised by consumer organisations for potentially misleading consumers, for instance when a product contained only 15% whole grain by weight, and considerably more added sugar [21] without any indication front-of-pack. The definition working group agreed that the mentioning of whole grain front-of-pack should only be allowed when a dietarily or nutritionally meaningful amount was present. Based on the following considerations, the level of 25% whole grain based on dry weight was chosen:

- The first whole grain food recommendation was based on the United States Dietary Guidelines for Americans, including the guidance to make ‘half your grains whole’. The first whole grain health claim allowed by the U.S. FDA defined a whole-grain product eligible for a claim around whole-grain bread with 51% whole-grain wheat flour [37]. Because 1 slice (serving) of whole-wheat bread has 16 g whole grain, one-half of the full serving of whole grain would be 8 g. The 8 g whole grain, which was used in most early epidemiological studies examining the relationship between whole grain and health outcomes, was considered as the minimum meaningful amount of whole grain deserving of mention front-of-pack.
- This 8 g whole grain/serving is currently widely recommended as a minimum level, for example, in the regulation in Malaysia [18], the recommendation by the UK-based Institute of Grocery Distribution [28], the whole grain stamp systems outlined below of the Whole Grains Council (WGC) [27], and the Australian Grains & Legumes Nutrition Council (GLNC) [38]. The GLNC states the following: *To carry GLNC certification a product must (…) contain at least 8 g whole grain per manufacturer serve AND at least 25% whole grain ingredients.* For the WGC, 8 g per serving or 25 g/100 g (depending on the country) is the minimum level for using a stamp, whereas for calling a food a whole-grain food, at least half of the grain ingredients have to be whole. The stamps indicate both the % whole grain of all grain components and the amount of whole grain per serving or per 100 g. The whole grain intake recommendation of 48 g is the extrapolation of the average number of servings of grain per day in the U.S. (6 servings), and if each serving delivers 8 g whole grain, then the daily recommendation is 48 g whole grain.
- When using the relatively small serving sizes as recommended by the USDA and the Whole Grains Council [27], and following the criteria of the GNLC, 8 g whole grain per serving corresponds to ~25–30 g whole grain/100 g.
- Serves or serving sizes are defined differently in different countries. Therefore, in order to avoid confusion in a global definition, the minimum amount of whole-grain ingredients is expressed as a percentage.

Finally, the combination of at least 50% whole-grain ingredients for whole-grain foods and at least 25% for inclusion of “whole grain” in front-of-pack labelling is in line with regulations, recommendations by Codex, and other authoritative bodies. These organisations require two levels of a nutrient in a food (such as “source of” and “high” dietary fibre), where the amount required for the “high” qualification is twice the amount required for “source of”. For whole-grain foods we propose that there is application of the same approach as complying with the ‘source of’ and ‘high in’ descriptors, which is unique. Currently, no precedent exists for applying regulatory standards associated with nutrients onto food groups. The dual levels will also address both the need for a high whole-grain level in whole-grain foods, as expressed by consumer organisations, and for a lower but meaningful minimum level for inclusion of whole grain in front-of-pack labelling to attract consumers not accustomed to higher levels of whole grains in food.

## 4. Final Remarks

Both the Whole Grain Ingredient and Whole-Grain Food definitions have been ratified by the leading international cereal science associations, including the C & G Association, the Healthgrain Forum, and the International Association for Cereal Science and Technology (ICC). The Whole Grain Initiative’s definitions working group, in collaboration with the Whole Grain Initiative leadership team, is working across countries and regulatory authorities to advocate for adoption of these definitions globally. There are numerous reasons why whole-grain intake remains low in most regions across the globe, and the members of the WGI are committed to reducing the barriers. The consensus definitions presented here seek to improve consumer confidence in the integrity of the label and help support consumers in selecting foods with evidence-based health benefits, while providing aspirational targets for food manufacturers wishing to describe their products as a “whole-grain food” and highlight the whole-grain content on labels. The adoption of the Whole Grain Initiative consensus whole grain definitions is strongly encouraged and should serve as a reference point for national regulatory authorities. 

## Supplementary Materials

The following are available online at: https://www.mdpi.com/article/10.3390/nu14010138/s1. Document S1, Definition of whole grain as food ingredient. Document S2, Definition of a whole-grain food. Document S3, Calculation of percentage of whole-grain ingredients based on dry weight.
