# Supplementary material for: Consensus, Global Definitions of Whole Grain as a Food Ingredient and of Whole-Grain Foods Presented on Behalf of the Whole Grain Initiative
Source: Nutrients. 2021 Dec 29;14(1):138. doi: 10.3390/nu14010138 (PMC8746580; doi:10.3390/nu14010138)
Supplement: Supplementary file 1 [file nutrients-14-00138-s001.zip › nutrients-1509213-supplementary.pdf]

Supplementary Materials

Supplementary document S1: Definition of whole grain as food ingredient. S2: Definition of a whole-grain food. S3: Calculation of percentage of whole-grain ingredients based on dry weight.

Supplementary document S1: **DEFINITION OF WHOLE GRAIN AS FOOD INGREDIENT**

> Whole Grain Initiative **Definition of Whole Grain as Food Ingredient**
> Whole grains shall consist of the intact, ground, cracked, flaked or otherwise processed kernel after the removal of inedible parts such as the hull and husk. All anatomical components, including the endosperm, germ, and bran must be present in the same relative proportions as in the intact kernel.[1]
>
> [1] The use of the term wholemeal may be legally protected in some jurisdictions and may be equivalent to whole grain. The use of this term should be checked within local contexts.

1) This definition applies to cereal grains in the Poaceae family, and pseudocereals listed in Annex 1, that are used for human consumption.

2) Processing of cereals and their fractions includes dry and wet methods which should be executed according to good manufacturing principles and considers the following points:

   a) A batch of grain consisting of one or more varieties or classes of a single species may be temporarily separated into fractions and considered whole grain if the fractions are recombined in the original proportions.

   b) Grain fractions from one or more varieties or classes of a single species that originated from different batches and combined to reflect the original proportions are considered whole grain.

   c) Small, generally unavoidable losses of components, that occur through processing consistent with safety and quality standards are allowed.

   d) Fermented, malted or sprouted grains containing all of the original bran, germ and endosperm shall be considered whole grains as long as nutrient values have not diminished; for malted or sprouted grains the length of the sprout should not exceed kernel length.

ANNEX 1.
Pseudocereals used for human consumption considered as grains

**Species**	Amaranth
	Buckwheat, Tartary buckwheat
	Quinoa

The anatomical components of pseudocereals, being dicotyledons, are different from those of the monocotyledonous cereal grains. As for cereal grains, all edible anatomical parts of processed pseudocereals must be present in the same relative proportions as in the intact seed.

APPENDIX TO THE DEFINITION OF WHOLE GRAIN AS FOOD INGREDIENT

This definition refers to whole grain as a raw material and a food ingredient. The definition is generic and does not include quantitative criteria relevant for a single grain. Such criteria are available in existing standards and specifications. The term kernel is used for many widely consumed grains, such as wheat, maize, rice, barley and rye. Other commonly used terms include seed, berry, groats and grain. Additional terms, both in English and other languages may be used as well. The anatomical components referred to in the definition are:

- The bran fraction including the pericarp (outer and inner pericarp), the seed coat and the aleurone layer of the cereal grain.
- The germ fraction.
- The endosperm fraction including starchy endosperm.

Ad 1. The Poaceae (also called Gramineae) family includes all kinds of edible and other grasses. A wide range of edible ones, called cereal grains, is listed in definitions (e.g. AACCI and Healthgrain) and by the Whole Grains Council (see https://wholegrainscouncil.org/whole-grains-101/whole-grains-z) together with the pseudo-cereals listed in Annex 1. The global definition allows addition of newly developed species of cereal grains, such as Tritordeum, when they are accepted by the relevant authoritative body as grain for human consumption. Following existing definitions and dietary guidelines of whole grain worldwide, pulses and legumes are not included.

Ad 2. Most grains need to be processed before consumption, which may include cleaning (removal of stones, stems, etc.), removing inedible parts (e.g., hull/husk), dry (e.g., milling) and wet (e.g., malting, sprouting, fermenting) processing to make nutrients more available and improve palatability, and stabilizing (e.g., toasting germ and rice bran) to inactivate enzymes that reduce storage stability. Therefore, in addition to the "ground, cracked, flaked" mentioned in the AACCI and Healthgrain definition, "otherwise processed" is included. Issues related to further processing such as baking and extrusion for preparing food products are outside the scope of the definition of whole grain as a food ingredient.

Ad 2b. In most commonly applied milling processes endosperm, bran and germ are separated for later recombination. For most whole grains and flours that require a long shelf life, the germ and bran fraction are heat stabilized, followed by recombination with the endosperm of a batch of grain that entered the plant later. In many large flour milling plants, a wide range of varieties of the same grain are processed.

Ad 2c. Consistent with good standards of manufacturing practices, small, generally unavoidable losses resulting from removal of the hull/husk, milling, or processing (e.g., minimally processed bulgur and nixtamalized maize), as well as minimal removal of outer layers are acceptable. Allowable limits for the percentage removed should be evidence-based, be kept to a minimum, may depend on the specific grain type or variety, and on local regulations or constraints (e.g., in some jurisdictions 2% is the maximum loss allowed for wheat).

Ad 2d. The Global Working Group acknowledged that current practices in grain processing include methods such as sprouting and fermentation and agreed with the text of item 2d – the AACCI statement for malted and sprouted grains (2008), with addition of 'fermented', since processing increasingly includes fermentation of grains, flours, or a fraction (e.g. bran, where bakeries apply long partial pre-fermentations before reconstitution to a whole grain dough for 'standard' fermentation. The Global Working Group also agreed with the recommendation by the Healthgrain Forum (2017) that processing should not result in a >10 % reduction in the dietary fibre content (as an indicator of the amount of beneficial components within the whole grain).

Supplementary Document S2: **DEFINITION OF A WHOLE-GRAIN FOOD**

Whole Grain Initiative **DEFINITION OF A WHOLE GRAIN FOOD**
INCLUDING REQUIREMENTS FOR DESIGNATING WHOLE GRAIN FRONT-OF-PACK

I. Definition of a whole-grain food

A whole-grain food shall contain at least 50% whole-grain ingredients based on dry weight

II. Requirements for designating the presence of 'whole grain' front-of-pack

Foods containing a minimum of 25% whole-grain ingredients based on dry weight, may make a front-of-pack claim on the presence of whole grain but cannot be designated 'whole grain' in the product name.*

*The decision to include "and at least 8 grams / serving" in addition to "a minimum of 25% whole-grain ingredients based on dry weight" should be left to national authorities.

The following remarks will apply for I. and II.

- Whole-grain ingredients used to make whole-grain foods defined in **I.** and **II.** must conform to the Definition of Whole Grain as Food Ingredient issued by the Whole Grain Initiative[1]
- The dry weight of a food or ingredient is the weight of the food after its moisture content has been subtracted from its total weight. The content of whole grain is the dry weight provided by all whole-grain ingredients expressed as a percentage of the total dry weight of the food product.
- Reporting the percentage of whole grain in a product in any front-of-pack labelling is strongly recommended, for ensuring fair practices in the food trade and ease of consumer comparison among and between products.
- National regulations and definitions, if requiring a greater proportion of whole grains in a product, will prevail. In countries with existing definitions for whole-grain foods that permit less than 50% for labelling a product as a 'whole-grain food', the adoption of the proposed definition is strongly encouraged to promote consumption of whole grains, to provide consistent messaging, and improve public health.
- Criteria involving permitted levels of other nutrients, ingredients or healthy eating criteria are the responsibility of national authorities. Linking of whole grain labelling only to foods that are considered part of a healthy diet is recommended.
- A standardised global definition for whole-grain foods will help consumers make informed choices, give food manufacturers clear guidelines for formulation and labelling, and provide a uniform basis for nutrition and health research.

DEFINITION OF A WHOLE-GRAIN FOOD ANNEX – Explanatory Notes

General remarks

- The definition is generic and does not include quantitative criteria for specific types of products.
- The terms 'whole grain' and 'whole-grain food' are generic and actual information that would appear on package will most often be product specific, for example: 'whole-grain bread', 'whole-grain pasta', 'whole-grain cereal'.
- The use of the term wholemeal may be legally protected in some jurisdictions and may be equivalent to whole grain. The use of this term should be checked within local contexts.
- The definition requires whole-grain foods to contain on a dry-weight basis more total whole-grain ingredients than any other food ingredient and aims to contribute to credibility for consumers.
- As a result of the inclusion of 'based on dry weight', whole-grain products with a high-water content (such as a ready-to-eat porridge or fresh pasta) can be labelled as a whole-grain food.
- 8 grams whole grain per serving – for common serving sizes equivalent to 25-30% whole grain on a dry-weight basis - is widely considered as the minimum contribution of a dietarily meaningful amount of whole grain.
- Serving sizes have different definitions in different countries. Therefore, in order to avoid confusion, the minimum amount of whole grain is expressed in **II.** as a percentage. Use of serving sizes in labelling and communication is optional and seen to be a mechanism for consumer facing communication rather than a basis for a global regulation.
- National authorities may choose to add "and at least 8 grams / serving" to the text of **II**.
- Dietary intake of whole grains is associated with improved health and the purpose of this definition is to help consumers identify and choose healthier foods based on their whole-grain content.

Calculation of percentage of whole-grain ingredients based on dry weight

The percentage of whole grain based on dry weight to be used for the definition may be based on either an analysis or a calculation from known or factual average values for the ingredients in the product. The calculation may also be based on commonly determined and accepted data for the ingredients, such as data in food composition databases.

Labelling

The percentage of whole grain to be designated on the pack should be based on local regulations, such as the widely used Quantitative Ingredient Declaration system, also included in the Codex Standard 1 – 1985, section 5.1: Quantitative ingredients declaration. In particular for high-moisture products the percentage of whole grain based on dry weight will be higher than the percentage based on QUID.
In whole-grain foods as defined in **I.** the percentage of whole grain may vary considerably: a whole-grain food may contain at least 50% up to 100% whole-grain ingredients. For this reason, it is recommended to have front-of-pack labelling that assists consumers choose based on whole-grain content and encourages industry to increase the proportion of whole-grain ingredients in their product.

Further explanations are provided in the document *Whole-grain food definition. Additional Information and guidance* - available at the Whole Grain Initiative website (http://www.wholegraininitiative.org).

Supplementary document S3:

Calculation of percentage of whole-grain ingredients based on dry weight

(Example given in Appendix 4 of the Whole Grain User Manual, Denmark)

Formula for dry matter calculation – introduction

- The content of whole grain calculated as the product's dry matter is the weight of the dry matter provided by all whole-grain ingredients expressed as a percentage of the total weight of dry matter in the final product.
- The quantity of dry matter is taken from average values based on either analysis, calculation from known or factual average values for the ingredients in the product, or calculation on the basis of commonly determined and accepted data for the ingredients.
- Flour and cereal products are the main ingredients in bread. In order to calculate the whole-grain content of bread based on its dry matter, a standard value of 15% for the water content of the flour and cereal can be applied (as long as the water content does not exceed 15%). In other words, these ingredients have a dry matter value of 85%. The bread's remaining ingredients have other dry matter values. For example, seeds have a lower and vegetables a higher water content.

Recipe for a hypothetical bread:

351 g	Whole grain wheat flour
50 g	Sunflower seeds
200 g	Wheat flour
300 g	Water
50 g	Yeast
9 g	Salt
40 g	Oil
**1,000 g**	**Ready dough in total**

(the loaf weighs 900 g after baking due to evaporation during the baking process, based on a standard evaporation of 10%).

Percentage of whole-grain ingredients calculated on the basis of dry weight– generic formula:

[(Whole grain ingredients x 0.85) x 100%] :
[(Whole grain ingredients + other flour and cereal ingredients) x 0.85
+ all other ingredients multiplied by their dry matter values]

Specific formula for the hypothetical recipe. Percentage of whole-grain content = 51%

[Whole grain ingredients dry matter (351g x 0.85%=) **291 g** x 100 **:**
Total dry matter: 574g

**[** (351 g whole grain wheat flour + 200 g wheat flour) x 0.85%)	**468 g**
+ (50 g sunflower seeds x dry matter value for these seeds 95%)	**47,5 g**
+ (50 g yeast x dry matter value for yeast 19%)	**9,5 g**
+ (9 g salt x dry matter value for salt 100%)	**9 g**
+ (40 g oil x dry matter value for oil 100%)	**40 g]**

Calculation of the percentage of whole grain for the Quantitative Ingredient Declaration

QUID is the relation between the weight of all whole grain ingredients and the weight of the final product, expressed as a percentage, to be mentioned as required by regulations, on the pack.

The hypothetical bread contains 39% whole grain:

[351 g (whole grain wheat flour)] x 100 **:** [900 g (weight of bread after baking)] = **39%**
